# An idea to explore: Interdisciplinary capstone courses in biomedical and life science education

**DOI:** 10.1002/bmb.21673

**Published:** 2022-10-03

**Authors:** Pauline M. Ross, Lucy Mercer‐Mapstone, Liana E. Pozza, Philip Poronnik, Tina Hinton, Damien J. Field

**Affiliations:** ^1^ School of Life and Environmental Sciences University of Sydney Camperdown New South Wales Australia; ^2^ Faculty of Science University of Sydney Camperdown New South Wales Australia; ^3^ FHM MediaLab, Education Innovation, School of Medical Science, Faculty of Medicine and Health The University of Sydney New South Wales Australia; ^4^ Sydney Pharmacy School, Faculty of Medicine and Health The University of Sydney New South Wales Australia

**Keywords:** biomedical and life sciences, capstone, curriculum design, interdisciplinary capstone

## Abstract

While biomedical and life science research have embraced interdisciplinarity as the means to solving pressing 21st century complex challenges, interdisciplinarity in undergraduate education has been more difficult to implement. As a consequence, disciplinary rather than interdisciplinary capstones have become ubiquitous. Disciplinary capstones are valuable for students because they enable them to integrate knowledge and skills within the discipline, but they are also limiting because the integration is within rather than across disciplines. In contrast to a capstone, which involves a single discipline, interdisciplinary capstones require two or more disciplines to combine and integrate across disciplinary boundaries. Interdisciplinarity, where two of more disciplines come together, is difficult to implement in the biomedical and life science curricula because student majors and finances are administered in ways, which reinforce institutional organization of schools and faculties and prevent collaboration. Here in this “idea to explore” we provide an interdisciplinary capstone model where students enroll in disciplinary courses, but then these disciplinary courses and students collaborate on interdisciplinary real‐world problems. This interdisciplinary capstone model was implemented across two diverse and large biomedical and life science schools within two faculties in a research intensive, metropolitan university. This approach allows for integration of the biomedical, social and ethical perspectives required when solving problems in the real world, such as COVID‐19. Interdisciplinary learning also better prepares students for higher degree research and future careers. Overcoming disciplinary curriculum silos and faculty barriers is critical if we are to meet expectations of acquiring interdisciplinarity as a key competency.

## Graphical abstract

Comparison between disciplinary and multidisciplinary silos and interdisciplinary learning to solve real world problems.

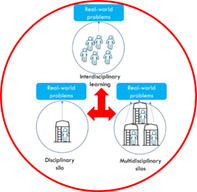

## 1 INTRODUCTION

Interdisciplinary learning has increasingly been seen as necessary yet challenging to implement in undergraduate biomedical and life science education.^1^, ^2^, ^3^ Interdisciplinarity emerged as researchers recognized that solving the complex challenges of our time, such as the spread of infectious diseases, food and water security and climate change, requires the integration of expertise and skills of several disciplines and sub disciplines and that breakthroughs often occur at these intersections.^4^, ^5^, ^6^, ^7^, ^8^ This has been brought into sharp focus with the COVID‐19 pandemic.

Interdisciplinarity has been defined when two or more disciplines combine and interact across disciplinary boundaries to solve a problem, creating a solution that cannot be produced by one discipline alone. Boix Mansilla^9^ described it as “the capacity to integrate knowledge and modes of thinking in two or more disciplines to produce a cognitive advancement, for example, explaining a phenomenon, solving a problem, creating a product, raising a new question in ways that would have been unlikely through single disciplinary means” (Boix Mansilla, p.4). Tripp and Shortlidge^3^ defined it “… as the collaborative process of integrating knowledge/expertise from trained individuals of two or more disciplines leveraging various perspectives, approaches and research methods/methodologies to provide advancement beyond the scope of one discipline's ability”.^2^, ^3^, ^9^

Interdisciplinarity has been more difficult to implement in undergraduate education than in research because student discipline concentrations and finances administered by schools and faculties reinforce organization structures and prevent collaboration. As a consequence, disciplinary rather than interdisciplinary capstones have become ubiquitous. Capstone experiences, often termed ‘culminating experiences’, have become popular in a wide range of undergraduate degrees because they provide opportunities for students to connect and integrate knowledge and skills of the biomedical and life science disciplines, attain graduate qualities, and transition into professional pathways and employment.^10^, ^11^, ^12^, ^13^ While disciplinary capstones are valuable, they are also limiting because the integration is within rather than across disciplines. In contrast, interdisciplinary capstone courses integrate content within and outside the biomedical and life science disciplines and provide opportunities for students to use their disciplinary knowledge to solve real problems that dominate life after university.^14^ Creating true interdisciplinary capstone experiences requires faculty and students from two or more disciplines across faculties and schools to merge and collaborate.

In this article we describe a curriculum model for implementing interdisciplinary final year capstone experience in biomedical and life science undergraduate education where students from different disciplinary backgrounds come together to work in interdisciplinary teams to solve 21st century problems. To do this we first describe the history of the emergence of interdisciplinarity. Second, we critically analyze interdisciplinary capstones which have so far been implemented. Finally, we describe a capstone model of interdisciplinary learning and assessment and the challenges faced in design and implementation and thfor interest present some preliminary comments from students.

### 1.1 History of the emergence of interdisciplinarity

Interdisciplinary approaches to biology research and education emerged when the report ‘BIO2010: Transforming Undergraduate Education for Future Research Biologists’^7^ recommended embedding interdisciplinary learning into the biology undergraduate curriculum. This was subsequently supported by a series of publications and the National Research Council (NRC) in its 2009 publication of “*A New Biology for the Twenty‐First Century*”.^8^, ^15^, ^16^, ^17^ It was the Vision and Change in Undergraduate Biology Education statement,^17^ however, which consolidated and advanced the importance of biological concepts being applied to real world problems.^18^ AAAS^17^ identified the interdisciplinary nature of science as a core competency for students. For example, the management of habitats such as coral reefs and diseases such as COVID‐19 requires the consideration of biomedical, biological, political, economic, sociological, and ethical dimensions.^19^

Interdisciplinary learning, through complex problem solving, was seen as a way for undergraduate students to use their disciplinary knowledge in real world contexts, allowing them to become aware of disciplinary affordances and limitations and to be much better prepared for the workplace.^20^, ^21^, ^22^, ^23^, ^24^ Interdisciplinary experiences develop complex problem solving, collaborative, and transferrable skills.^22^, ^25^, ^26^ Moreover, DeZure^25^ described a comprehensive set of benefits of interdisciplinary learning, emphasizing the dynamic changes which occur in knowledge construction when disciplinary boundaries are crossed and the shift in perspectives when integration of disciplines occurs to solve pressing social and scientific challenges.

### 1.2 Approaches to interdisciplinarity

Approaches to interdisciplinary education vary widely (Table 1). Table 1 provides a summary of recent curriculum designs of interdisciplinary capstones, including a description of the design, the degree of interdisciplinary integration and collaboration and the pedagogy used. The degree of integration in interdisciplinary capstones has ranged from traditional (students from the same discipline taking on roles) to overlapping and fully integrated (students working in mixed interdisciplinary groups). Most commonly interdisciplinary learning is organized around students collaborating in project groups to solve a real‐world problem (e.g., References [22, 24, 26, 27]) where students take on different disciplinary roles (e.g., References [22, 23, 28], Table 1).

**TABLE 1:** Summary of recent studies on interdisciplinary capstones, including a description of the design, the degree of interdisciplinary integration and collaboration and the pedagogy used

Study	Description of design	Degree of integration	Collaboration across disciplines	Pedagogy
Anderson et al.^22^	An interdisciplinary project for students to achieve learning outcomes in eutrophication. Students read about interdisciplinary work and a management plan and write an essay on whether the plan had achieved the interdisciplinary aim. Students used research methods in ecology and environmental science, and their own disciplinary backgrounds	Traditional Problem was integrative and students considered different disciplines as humans play a role in management action	Students were allocated roles of phycologist, microbiologist, agronomist, limnologist had variety of year grades from first to senior	Active, case‐based learning
Everingham et al.^26^	An interdisciplinary compulsory first year unit. Purpose of intervention to reduce mathematics anxiety and improve student learning outcomes Students were taught through interdisciplinary problems, and mathematics in these problems highlighted. Students enrolled in the unit were guided using a framework of interaction, assessment, relevancy and technology.	Overlapping Content of mathematics was taught through integrative problems. Considered interdisciplinary because students were from different majors	Students were from majors in biology and environmental and Earth sciences and a small proportion of Mathematics and Physics.	Active, case‐based learning
Kulcsar et al.^20^	An interdisciplinary graduate course in natural resource management, “Water and Society” a 5‐year project (2006–2013) with support from National Science Foundation. Discipline‐specific components demonstrating what the other disciplines could do to help solve the problem. Students exposed to various stakeholder interests and specific lectures to deepen students' knowledge in the various fields represented in the course. Faculty guidance intensive in first few weeks, but reduced later in semester to enable students to extend their knowledge themselves Students developed individual diagram of water system based on their own disciplinary training and background. Students then worked in groups to create a collective understanding of issue, incorporating all disciplinary perspectives.	Fully integrated Faculty were from five discipline fields.	Student were from different disciplines, which varied each year. For example: Agricultural economics, Agronomy, Civil Engineering, Landscape, Architecture, Sociology, Political Science	Active, problem‐based learning using methods in constructivist teaching
Laflamme et al.^24^	An advanced interdisciplinary research laboratory (AIRLab). Project: students were to design a vessel that would allow for continuous monitoring of biological, chemical and geological parameters related to decay and fossilization. Students in interdisciplinary groups complete a grand challenge to create a continuous monitoring of biological, chemical, mineralogical changes during the decay process. Students were required to conduct thorough analysis of the literature and reflection allowed students to understand the limitations of disciplinary knowledge. Students use their disciplinary knowledge, but educational developer was available to discuss approaches Used KLSI (Kolb Learning Style Inventory) methodology to help	Overlapping Problem was integrative and students used discipline knowledge to contribute to project storyboard and tasks. Team leader changes monthly	Students were from chemistry, physics, biology and earth sciences	Active learning, DEAL method (Describe, Examine, Articulate and Learning) also Reflection
Olechnowski^23^	An interdisciplinary capstone project. Purpose to provide students with an interdisciplinary understanding of the nature of Conservation Biology and Wildlife Management. Students designed an interdisciplinary capstone management plan. Students were required to take lecture material into account, while also conducting their own research on the issue. Students used research methods in typical of Conservation Biology journal	Traditional Problem was integrative and students considered different disciplines as humans play a role in management action	Students were allocated roles depending on management issue—for example, conservation biologist, ornithologist, member of PETA, bioethicist	Active, project‐based learning
Spelt et al.^28^	Interdisciplinary master course, in science and engineering. Evaluation of cognitive, emotional, social student learning outcomes. Students reported disciplinary knowledge in interdisciplinary thinking using a reflective journal. Students used reflection to gain understanding of interdisciplinary thinking.	Fully in integrated Students shared knowledge on interdisciplinary research, called ‘learning communities’. Interdisciplinary teaching group.	Students were in an interdisciplinary masters, natural and social, engineering students	Problem solving, integrative framework used
Watson et al.^27^	Problem‐based capstone learning in microbiology where students partnered with a local farm, a community garden, and a free clinic to address problems experiences by these partners. Course design with Merrill's (2002) First Principles of Instruction model in mind – 4 phases 1. Activation of old knowledge 2. Demonstration of skills, 3. Application of skills 4. Integration of skills to real‐world activity Students needed disciplinary expertise to answer questions Students kept laboratory notebooks and received guidance throughout process	Overlapping Students wrote up proposal, which was assessed and integrated into a group proposal	Students from microbiology agroecology and molecular biology	Active, problem‐ and service‐based and reflection

*Note*: Degree of integration ranged from traditional (students from the same discipline taking on roles) to overlapping and fully integrated (students working in mixed interdisciplinary groups).

Interdisciplinary learning is less frequently organized around students with different disciplinary backgrounds working together in authentic interdisciplinary teams taught by interdisciplinary faculty (e.g., References [20, 26, 29], Table 1). Pedagogies are generally active learning approaches^2^, ^22^, ^23^, ^26^, ^29^, ^30^ such as problem solving,^27^, ^28^ project work,^24^, ^27^ case studies,^22^, ^26^ technology and demonstrations with guest lectures^26^, ^29^ and constructivism.^20^

To guide interdisciplinary learning, frameworks such as the Interdisciplinary Science Framework (IDSF) have been created through conversations with researchers.^3^ The IDSF provides a set of ingredients essential to interdisciplinary learning, including a basic understanding of disciplines (disciplinary grounding), how disciplines integrate (advancement through integration), the use of different disciplinary research methods, and collaboration across disciplines. Assessment frameworks from Boix Mansilla et al.^31^ which outline criteria and standards that can be shared with students and faculties also answer questions on how to measure the performance of students in interdisciplinary learning.

Despite interdisciplinary learning now being at the leading edge of biomedical and life sciences undergraduate education, barriers remain in its implementation.^3^, ^7^, ^8^, ^15^, ^17^, ^32^ This is in part because universities reinforce disciplinary boundaries in both research and education,^3^ but also because many biomedical and life science faculty are ill equipped to teach interdisciplinary skills. Departments mostly seek, hire, and promote faculty who are experts in their discipline with an academic identity and allegiances to disciplines being formed during undergraduate studies and cemented during PhD and postdoctoral training.^3^, ^33^ Undergraduate biomedical and life sciences curricula induct students into their discipline in the same way as their faculty teachers—through a narrowing of disciplinary focus and expertise over the course of degree. This means that students are often unfamiliar and ill‐equipped, and not sufficiently supported, to apply their disciplinary knowledge in interdisciplinary settings. If we are to meet the challenge of developing students' competency in interdisciplinarity then we need to go beyond students assuming hypothetical disciplinary roles and create models which bring together students from different disciplinary backgrounds to solve real‐world problems.

### 1.3 Interdisciplinary capstone model and challenges

**FIGURE 1:**
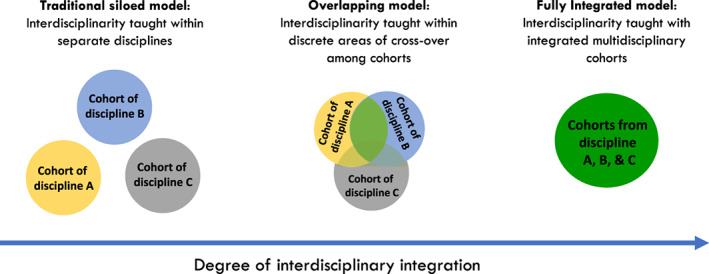
Degree of interdisciplinary integration

**FIGURE 2:**
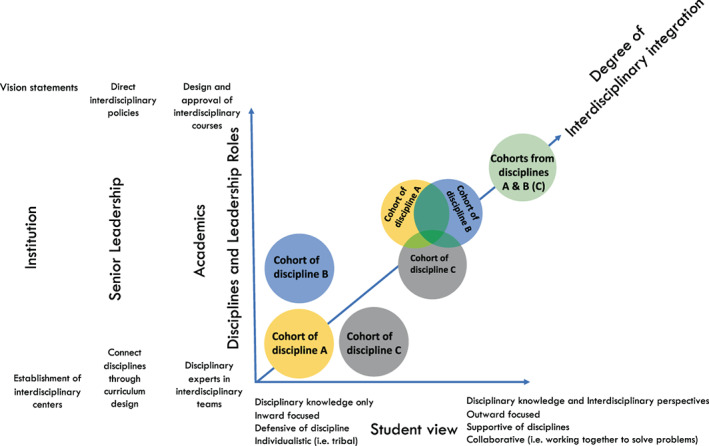
Degree of interdisciplinary integration and actors involved in the implementation including academics and senior leaders in the institution

We created and embedded a curriculum model of interdisciplinarity in each science concentration in a Faculty of Science. We defined “concentration” as a specific area or field of study within the broad field of life and medical science i.e. genetics and physiology. In our situation in Australia, the word “major” is used to encompass both broad and more narrower areas of study i.e. is inclusive of genetics, physiology, biology or medical science. We used the term concentration because it is a term more commonly used in curriculum across higher education. To create an interdisciplinary capstone course in the junior or final year of medical and life science degree, each concentration formed a partnership with at least one or more other concentration to form an interdisciplinary capstone. Thus, combined capstones effectively had students from at least two concentrations or two distinct disciplines (Figure 1). Figure 1 provides a conceptualisation of the degree of interdisciplinary integration from a traditional siloed model to an overlapping model where there is both disciplinary content and areas of interdisciplinarity or cross over among cohorts, to a fully integrated model. For example, an interdisciplinary capstone in biochemistry and molecular biology formed a two‐way partnership with mathematics concentrations while infectious diseases formed a three‐way partnership with biology and history and philosophy concentrations (i.e., overlapping model in Figure 1). Partnerships between and among courses were required to have at least 50% disciplinary and 50% interdisciplinary content and activities. Most adopted the overlapping model whereby two or three capstone courses combined for a team‐based interdisciplinary project for 50% and maintained 50% discipline‐specific content for cohorts separately (Figure 1, A and B or A, B and C courses combined). Less frequently adopted was an integrated model where capstones effectively became one coherent interdisciplinary capstone where interdisciplinary content was dominant (Figure 1). Initially the overall model of interdisciplinarity for the curriculum was written and described by presidents in vision statements, created in policy by senior leaders and then designed and realized by staff in faculties and at the coal face (Figure 2). Figure 2 provides a conceptualisation between the degree of interdisciplinary integration as experienced by the student and the agents involved in the design and implementation including academics and senior leaders in the institution.

Some course partnerships were formed between concentrations to accommodate certain restrictions such as cohort size disparity. The variation in cohort size between courses (ranging from less than five to over 300) was the result of students' preferences for particular concentrations. The disparity of student class sizes was resolved by either merging concentrations where there were small student cohorts or diversifying projects, where there were large student cohorts in concentrations. For example, small student numbers in two concentrations of, similar disciplines, such as food and nutrition, were often merged to act as one concentration to partner with a second distinct concentration such as statistics. Where there were large student cohorts, multiple partnerships were formed, working on a diversity of projects. Having comparable cohort sizes within partnerships was desirable, so that the formation of student groups with representatives of each of the disciplines could occur.

Further, as interdisciplinary courses were required for each concentration, students taking concentrations of both biology and genetics could be affected by the partnership approach. Students taking two concentrations, or two majors, were required to complete two interdisciplinary courses, so long as these courses were not in the same partnership. To ensure that these students were not affected, extensive cross‐checking of enrolments by concentration pathways had to be undertaken. For those students who found themselves requiring both interdisciplinary courses for concentrations which had partnered, other options (offered centrally by the university) were counted as an interdisciplinary experience for one of these concentrations.

This interdisciplinary capstone model was more authentic than other interdisciplinary curriculum models because students represented the discipline of their concentration, rather than taking on a role of a discipline. It also overcame administration and financial challenges. Given that student fees routinely follow the disciplinary or concentration pathways of student choice, constructing interdisciplinary capstones within concentrations meant that student enrolments and fees still went to the department or faculty within which the student was enrolled, which solved the problem of competition for students and funding.

**FIGURE 3:**
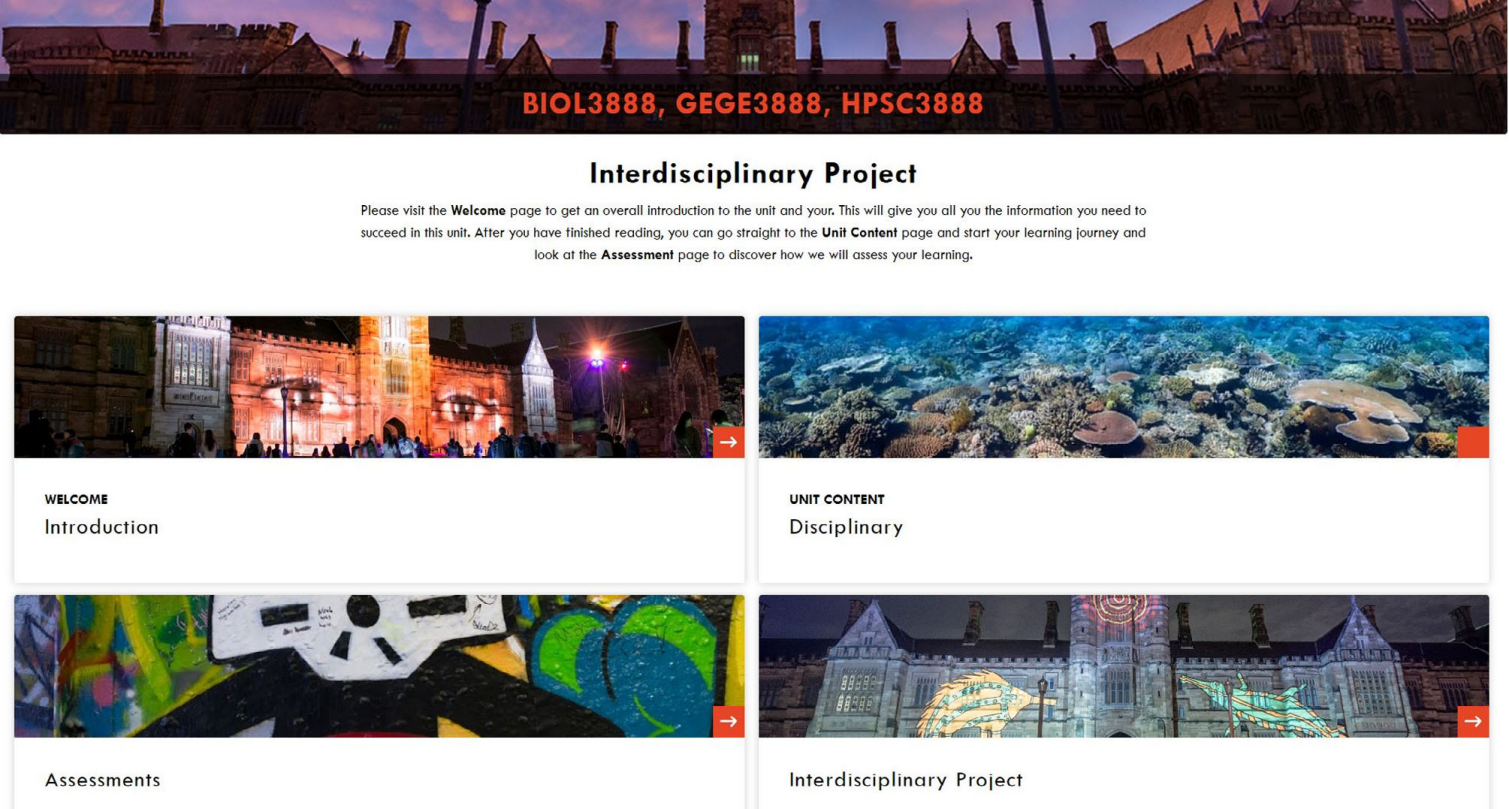
CANVAS LMS interdisciplinary site for biology, genetics and history and philosophy of science—a three way partnership

Administering combined cohorts from different capstones which would normally have separate CANVAS Learning Management System (LMS) sites was solved by a single LMS which enrolled students from all partnered course cohorts. This single LMS was a place where students and staff were able to source all the information and materials related to the interdisciplinary aspects of the course. On this site, there was clear signposting of discipline‐specific and interdisciplinary content and assessment (Figure 3). This has the added benefit of enabling students to see all the discipline content across courses, should they be interested in or wish to understand different disciplinary perspectives on their projects.

While faculty were willing to cross disciplinary boundaries to do interdisciplinary research, some were reluctant to cross the same disciplinary boundaries to do interdisciplinary teaching. First, faculty expressed anxiety about their inexperience in teaching and supporting students to develop the skills required for interdisciplinary work. Other studies have found that faculty may be reluctant to teach interdisciplinarity believing learning disciplinary knowledge and skills are sufficient.^34^ Solutions to this included a sustained program of support and academic professional development. Second, faculty also expressed anxiety about the ‘watering down’ of discipline concentration content. Typical complaints included “shouldn't final‐year capstones be focused solely on discipline‐specific research?”, aligning with the Kift et al.^12^ narrowed ‘mountain‐top’ conceptualization of capstone subjects which has historically been popular. To address these concerns, a consensus was reached where the minimum of interdisciplinary learning was set at 50% reflecting the overlapping model of integration and the other 50% being disciplinary content. Many courses did opt to increase the interdisciplinary proportion and did so predominantly by designing a more fully integrated delivery of disciplinary and interdisciplinary aspects (Figure 1). This ensured, as intended, that the concentration content acted as a foundation upon which to build and apply skills in interdisciplinary contexts, teams, and problems.

Assessment was, as expected in an assessment‐driven curriculum, the place where the largest number of issues weras raised. Other studies have found that assessment of interdisciplinary learning commonly involves group outputs such as management plans and grant proposals, developed through multiple stages of drafting and feedback,^20^, ^23^ often accompanied by individual reflection statements.^20^, ^27^, ^28^ Assessment associated with the interdisciplinary component of the capstones—a large interdisciplinary project – was aligned and shared between partnered courses (Table 2). We designed from the outset that the assessment in these interdisciplinary capstone courses be aligned so that the learning outcomes of interdisciplinary projects were validly evaluated. This also had the effect of team members being accountable to one another – as in any project‐based work environment.

**TABLE 2:** Shared assessment scheme adopted across interdisciplinary courses

Focus of assessment	Assessment item	Weighting (%)	Type
Disciplinary (distinct)	Assessment at coordinators discretion	20	Individual
	Disciplinary exam	30	Individual
Interdisciplinary (shared)	Project report/output	30	Group
	Project oral presentation	20	Group
	Written reflection^a^	0	Individual
	Self‐ and peer‐evaluation of groupwork^b^	0	Individual

*Note*: Mostly this was 50%:50% disciplinary and interdisciplinary content for both individual and group work.

^a^ Sometimes weighted at 10% and increased the interdisciplinary project total to 50% and decreased the disciplinary exam to 20%. Some units removed the disciplinary exam entirely. When this happened the project report and oral presentation increased by 10%, giving a total for interdisciplinary content of 70%.

^b^ Could have been used as a factor to moderate the group assessment.

The usual concerns were consequently raised regarding how to grade a student's ‘true’ abilities given a high proportion of group work. The perception existed that group work problematically results in historically lower‐performing students receiving higher marks when working with historically higher‐performing students. Assessment was organized so that there was a minimum of 50% individually assessed tasks and 50% group work and most commonly a 50%:50% disciplinary to interdisciplinary break down, although this could be varied depending on the approach and although rare, there was flexibility to increase the interdisciplinary assessment component (see Table 2). Peer evaluations were used to assess group members, introducing an additional level of transparency and accountability around group marks occurred and sometimes was used to moderate the project, group report (Table 2).

Other courses within our institution which have a high proportion of group work have shown a trend towards students receiving a higher course mark than their personal average, potentially resulting in such courses being perceived as ‘an easy option’ by both staff and students. At this stage, however, those perceptions seem to be unfounded, based on a well‐designed curriculum to discourage such effects, and cannot with any certainty be causally linked to group work. It may instead be that the courses are designed using best practice evidence‐based approaches which allow space for a greater number of students to achieve at a higher standard, relative to other more traditional modes of teaching in science. Only time and more research will tell.

Some issues arose when some coordinators treated the 50% interdisciplinary and disciplinary concentrations streams of the courses as entirely separate when it came to assessment – rather than using one to support the other. Additional assessment burden could occur if coordinators broke up the disciplinary content and assessment into small components. For example, there were sometimes requests to break up the disciplinary exam into multiple, smaller quizzes. Most of these requests were unsuccessful, because there was a formal approval processes in place for assessment changes at both school and faculty levels and policies to prevent over assessing, through multiple low value tasks. Examples of such an integrated assessment scheme included coordinators using the disciplinary tasks to get students to conduct literature reviews on their own disciplinary area relevant to the interdisciplinary project, hence preparing them to contribute to their group's interdisciplinary research project. Most studies report positive student feedback on assessment in interdisciplinary learning.^22^, ^26^ Negative student feedback on interdisciplinary learning is more often related to students seeking more guidance from staff^24^ and frustrations when communication and technology breakdown occurs.^26^, ^28^

It is possible, as has been found by other studies, to identify criteria to measure interdisciplinary competency.^3^, ^31^ These criteria include disciplinary understanding, integration, perspective taking and collaboration. The interdisciplinary competence of students in this study was assessed through their group project work and individual reflection (Table 2). Project work by students working in groups accounted for 50% of the assessment, including a reflective statement. Reflective statements feature strongly as assessment components in interdisciplinary capstone courses.^20^ Literature on the assessment of student progress towards interdisciplinary competency is voluminous.^35^ Rubrics to assess interdisciplinary learning are abundant in the literature, however, these are only useful if they are clearly understood by both students and faculty. Common themes in the literature include the capacity of students to integrate and make connections across disciplines, identify differences in disciplinary perspectives and work in groups where assessment includes both self and peer assessment.^25^ To assess performance standards of interdisciplinarity we created, based on the literature, an interdisciplinary rubric (Table 3) which we attempted to implement. Interdisciplinary capacity can be described by performance standards using scalable verbs of what students do or how often a student demonstrates a skill (Table 3). It was much more straightforward to assess disciplinary content through assessment types such as exams, and written statements of project problems from a disciplinary perspective (Table 2) rather than interdisciplinary effectiveness which is a combination of integration, perspective taking and collaboration (Table 3).

**TABLE 3:** Rubric which describes competency in interdisciplinarity using four criteria and standards of attainment of the criterion of integration in terms using scalable verbs which articulate what students do and how often a student demonstrates the described verb

Criteria	1(P)	2(C)	3(D)	4(HD)
disciplinary understanding	Identifies disciplinary knowledge and uses it accurately and effectively in terms of concepts, theories, philosophical arguments and experimental design, to solve a problem, to explain a phenomenon or to create a product	Analyses disciplinary knowledge and uses it accurately and effectively in terms of concepts, theories, philosophical arguments and experimental design, to solve a problem, to explain a phenomenon or to create a product	Evaluates disciplinary knowledge and uses it accurately and effectively in terms of concepts, theories, philosophical arguments and experimental design, to solve a problem, to explain a phenomenon or to create a product	Critiques disciplinary knowledge and uses it accurately and effectively in terms of concepts, theories, philosophical arguments and experimental design, to solve a problem, to explain a phenomenon or to create a product
Integration	Identifies ideas and perspectives from two or more disciplines that culminates in an outcome.	Analyses ideas and perspectives from two or more disciplines that culminates in an outcome that would not have been possible with the use of one discipline alone	Evaluates ideas and perspectives from two or more disciplines that culminates in a creative outcome that would not have been possible with the use of one discipline alone	Critiques ideas and perspectives from two or more disciplines that culminates in a unique outcome that would not have been possible with the use of one discipline alone
Perspective taking	Identifies multiple viewpoints on a given topic including an appreciation of the differences between disciplines and their perspectives on how to approach a problem and their rules of evidence.	Analyses multiple viewpoints on a given topic including an appreciation of the differences between disciplines and their perspectives on how to approach a problem and their rules of evidence	Evaluates multiple viewpoints on a given topic including an appreciation of the differences between disciplines and their perspectives on how to approach a problem and their rules of evidence	Critiques multiple viewpoints on a given topic including an appreciation of the differences between disciplines and their perspectives on how to approach a problem and critiques their rules of evidence
Collaboration (cumulative)	Finds/usually shares knowledge and find common ground and creates a solution or product	Often shares knowledge, participates and contributes to decisions, respects differing points of view, adjusts to changes and creates a solution or product	Always shares knowledge, participates and contributes to decisions, respects differing points of view, adjusts to changes and creates a solution or product	Actively shares knowledge, participates and contributes to decisions, respects differing points of view, adjusts to changes and creates a solution or product

*Source*: Based on Boix Mansila et al.^3^

## 2 DISCUSSION

If students are to develop interdisciplinarity, then there needs to be curriculum designs which enable authentic integration of different disciplinary perspectives. The interdisciplinary capstone model we describe here enables students from different disciplinary backgrounds to integrate disciplinary understandings and skills to solve interdisciplinary problems. The unique feature of this model of interdisciplinary capstone courses in this idea to explore provide a description of “how” students from different disciplinary backgrounds and majors can be brought together in ways which are similar to what occurs in interdisciplinary research teams and in the workplace. Other approaches to interdisciplinary learning which have students from only one discipline take on roles of other disciplines, achieve limited authenticity,^3^, ^20^ (Table 1). This contrasts with more authentic examples of curriculum models in courses^36^ and as immersive and intensive challenges.^37^ Authentic integration of disciplines as described in this interdisciplinary capstone model brings together students and faculty from different disciplines or concentrations who have not previously interacted or collaborated on teaching or research. The key point of difference in this design and model of interdisciplinary capstone courses is that the students are representing different disciplines. This makes the design more authentic. It is then the interactions between students to solve problems, which creates integration and interdisciplinarity. In this way it aligns with the definition of Boix Mansilla (p.4)^9^ who described interdisciplinarity as “the capacity to integrate knowledge and modes of thinking in two or more disciplines to produce a cognitive advancement e.g., explaining a phenomenon, solving a problem, creating a product, raising a new question in ways that would have been unlikely through single disciplinary means”.

This is not, however, the only curriculum model for interdisciplinary learning. For example, Princeton's Integrated Science program, has a shared governance structure among faculties and departments thus seeking to overcome disciplinary silos. The National Experiment in Undergraduate Science Education (NEXUS) project similarly sought to better prepare pre‐medical students through the provision of a broader and more interdisciplinary curriculum.^29^ The project was a collaboration between four universities in the United States and focused on the development of modules integrating biological, chemical, physical, and mathematical sciences. To ensure the project came to fruition and maintained its focus, numerous levels of organization were established including a steering committee comprised of members of each participating university and an advisory board linked to broader stakeholders.

It is also important to acknowledge that Course Based Undergraduate Research Experiences (CUREs) where students work on original research problems have similarities and differences with this interdisciplinary model described here. CUREs, like interdisciplinary capstone courses aim to develop students critical thinking and problem‐solving skills.^38^, ^39^ In addition to these skills, and unlike CUREs, interdisciplinary capstone courses are focused on bringing together students and staff in concentrations who would not normally work together, including in some instances the disciplines of social sciences, mathematics and philosophy.

As others have found, interdisciplinary learning is easier to imagine than implement.^20^ The success of these interdisciplinary capstones was strengthened because there was institutional support from senior leaders of the university and faculty and interdisciplinary effectiveness was identified as a core graduate quality (Figure 2). A central education committee with representatives from all departments and faculties oversaw the process of design, approval and delivery. To co‐ordinate the implementation of the interdisciplinary capstone courses, a central faculty‐based staff member was also appointed who had mixed skill sets in science, interdisciplinary research, and education. The role of this staff member was to assist faculty to form partnerships between distinctly different science disciplines to ensure a truly interdisciplinary experience and to support and oversee the coherent implementation of the interdisciplinary capstone model. These interdisciplinary courses were supported and sustainable even through the COVID‐19 pandemic.

Although evaluation is still at the early stages, student evaluations of the units provide evidence that students have developed interdisciplinarity. For example, non‐solicited student responses commented on the benefits to work with students from other disciplines:

“The chance to work with other people in different disciplines has really helped me understand the concept of interdisciplinary group work and what some of the challenges may be with this” and “being able to work closely with my group was rewarding and challenged me to learn with an interdisciplinary mindset”.

and

“I developed my ability to work effectively with others studying in a different field(s) from me”.

Finally, the interdisciplinary capstone model described here resolved challenges and connected students in concentrations across a broad set of medical and life sciences to build the capacity of both students and staff to develop interdisciplinarity as a key competency. This approach allows for integration of the biomedical, social and ethical perspectives required when solving problems in the real world, such as COVID‐19. Interdisciplinary learning also better prepares students for higher degree research and future careers. Overcoming disciplinary curriculum silos and faculty barriers is critical if we are to meet expectations of acquiring interdisciplinarity as a key competency.
